# Clinical survival of No-prep indirect composite laminate veneers: a 7-year prospective case series study

**DOI:** 10.1186/s12903-023-02949-5

**Published:** 2023-05-03

**Authors:** Ozge Kam Hepdeniz, Ugur Burak Temel

**Affiliations:** Department of Restorative Dentistry, Faculty of Dentistry, Suleyman Demirel University, Isparta, Turkey

**Keywords:** Clinical trial, Indirect composite, Laminate veneer, Survival rate

## Abstract

**Background:**

The no preparation technique which is a reversible form of treatment characterized by the absence of tooth tissue preparation and preserves the soft tissue architecture while preserving all natural tooth structures is indicated in cases where the tooth structure allows material to be added. The aim of this study is to evaluate the clinical performance and survival rates of indirect composite laminate veneers with no preparation after 7 years.

**Materials and methods:**

A total of 80 indirect composite veneers were placed on maxillary anterior teeth in 35 patients (n = 80). Diastema (n = 64), wedge tooth anomalies (n = 9) and re-shaping (n = 7) were the main indications for veneer treatments. All laminate veneers were fabricated with an indirect microhybrid composite material (Gradia, GC Dental). No tooth preparation was performed. Light-cured resin cement (Choice 2, Bisco) was used to lute the veneers. Composite veneers were evaluated using Modified United States Public Health Service criteria. Survival rates of the veneers were calculated using Kaplan-Meier statistics. The data containing the results of the USPHS criteria at baseline, 2 years and 7 years was statistically analyzed using Wilcoxon Signed Rank test at the 0.05 level of significance.

**Results:**

The overall survival rate was 91.3%. After 7 years, 7 absolute failures including 4 debonding (marginal adaptation, score 4) and 3 fractures (fracture of restoration, score 3) were noted. Color match was scored as 1 (n = 34) and 2 (n = 15). Slightly rough surfaces (41 of 73 laminates) and slight marginal discoloration (15 of 73 laminates) were noted. The overall scores after 84 months were significantly higher than the baseline scores for the marginal adaptation (p = 0.008), color match (p = 0.000), marginal discoloration (p = 0.000), surface roughness (p = 0.000), and fracture of restoration (p = 0.001) criteria.

**Conclusions:**

In this study, indirect composite veneers without any preparation on maxillary anterior teeth showed acceptable performance in terms of both survival rate and quality of restorations. This procedure offers a predictable and successful treatment that ensures maximum preservation of the intact tooth.

## Background

Today, patients’ desire to have a healthy and harmonious tooth structure and an esthetically pleasing smile have revealed the necessity for considering the function and formation in dentistry, as well as the restoration of natural dental esthetics. For this reason, conservative treatments that can provide the patient’s expected outcome have been adopted as the first option in treatment [1, 2]. Laminate veneers are one of the conservative treatment options in this sense and also one of the most esthetically pleasing ways to achieve a more pleasant and beautiful smiles [3]. Laminate veneers allow us to change the tooth’s position, shape, size, and color by treating discolored, fractured, worn, and congenitally malformed teeth, diastemas, and esthetic abnormalities [4]. Direct and indirect techniques, including using various materials, have been used to fabricate laminate veneers [3]. Compared to direct laminate veneers, the high resistance to discoloration and fractures of indirect laminate veneers results in dentists preferring this technique [5]. It has been reported that, if a significant portion of the crown needs to be restored, the indirect technique may be recommended as it provides better abrasion resistance, proximal and occlusal contacts, less marginal leakage, and increased mechanical properties than direct techniques [6]. However, the necessity of using an adhesive cementation system, long chair time, and higher costs are factors limiting the application of indirect laminate veneer restorations [5]. Therefore, in deciding between direct and indirect treatment alternatives, the cost, along with social and timing factors, must be considered in addition to the esthetic and mechanical properties [1].

Among the esthetic restorative materials, clinicians have options ranging from composite resins to ceramics. Composite resin has long been the material of choice for both conservative and cosmetic procedures [7]. Composite laminate veneers are preferred due to the utilization of less invasive and more conservative treatment approaches to mask tooth discolorations, restore fractured teeth, and correct the unaesthetic tooth forms. However, wear, marginal fracture, and marginal discoloration are common problems of composite veneers and this causes a decrease in the esthetic outcome over time [5]. The substantial improvement in the bonding systems and physical properties of composite resins has greatly increased the success rate of laminate veneer treatments [8]. Clinicians also frequently use ceramic in laminate veneers because of its esthetically pleasing and durable properties. Similarly to composite veneers, ceramic veneers have limitations such as a fragile structure, tooth sensitivity, marginal defects, and restoration fracture [9]. A previous study reported that, although ceramics were a frequently preferred material due to their positive properties such as high fracture resistance and color stability, composites also produced excellent esthetic and mechanical results [10]. A randomized clinical trial, evaluating the short-term survival rate of indirect resin composite and ceramic laminate veneers, concluded that there was no statistically significant difference between the two materials in terms of clinical performance. However, surface quality changes were more common in the composite veneers and this was identified as a situation that may require further maintenance over time [11].

Today, preservation of the tooth structure with minimally invasive approaches is a necessity for successful restorations [12]. It is emphasized that the amount of remaining dental structure and the preparation design have considerable effects on the laminate veneers’ load failure [12]. The technique with no preparation (no-prep), which is characterized by the absence of tooth tissue preparation and its provisional phase, is a reversible form of treatment and maintains the architecture of the soft tissue by retaining all natural tooth structures. However, a lack of the need for anesthesia, absence of wear and postoperative sensitivity, no requirement of intermediate provisional restorations, minimal flexing stress, the long-term integrity of the margins, longer-lasting restorations, and higher levels of acceptance of treatment among patients are the other favorable features of no-prep laminate veneers [6, 12–15]. This type of treatment is indicated in cases where the tooth structure allows material to be added, including re-shaping, diastema closure, abfractions, and enlargement of the vestibular volume and incisal edge. This approach’s main contraindication is the fact that it cannot be used in cases that involve severe discoloration, deformity, and malposition, where the desired shape cannot be obtained by adding only restorative material without tooth preparation. In these cases, the need for preparation should be carefully evaluated [7]. Although no-prep veneers were considered the best option due to the maximum tooth structure preservation, this technique should be taken into account in terms of some potential limitations, including periodontal complications and esthetic outcomes such as gum inflammation as a consequence of over-contoured restorations [6, 7]. Therefore, in order to provide patients with the best in terms of esthetics, function, and longevity, a treatment plan with or without tooth preparation can be selected according to the patient’s clinical condition and demands [6].

The growing demand for having beautiful smiles with a more conservative treatment approach has increased interest in veneer restoration in anterior teeth [9]. In the literature, there is no information to date about the long-term survival rates of indirect composite laminate veneers without preparation. Therefore, this study’s aims were to evaluate the clinical performance of indirect composite laminate veneers without tooth preparation and determine the survival of composite laminate veneers. The study’s null hypothesis was laminate veneers without tooth preparation will show low survival rate and clinical performance in the long term.

## Material method

### Patient selection

The local ethics committee (2014/420) approved the protocol of this clinical study. All of the participating patients provided informed consent at the beginning of the study. Thirty-five patients (17 women, 18 men), ranging from 19- to 45-years-old, were included in the study. A detailed anamnesis and oral examination for each patient was performed. The inclusion criteria were as follows: having good general health, not allergic to resin-based materials, any pregnancy, having no active periodontal or pulpal diseases, being able to return for follow-up examination.

The patients with poor general health and oral hygiene, pregnancy, allergy to the resin-based materials, intense loss of tooth structure, hypoplasia, severe tetracycline staining, severe malocclusion, and parafunctional habits such as bruxism, anterior cross bite, and periodontal problems were excluded from the study. Existing root canal treatments, restorations, large cervical wedge-shaped defects, and existing restorations were further exclusion criteria determined by the study protocol. Patients with diastema, wedge tooth anomalies, and malformed teeth to be reshaped that could be corrected with additional composite without preparation were included.

### Restorative procedure

A single experienced operator with at least 10 years of experience in esthetic restorative dentistry and composite laminate veneers applied laminate veneers to 80 maxillary anterior teeth in 35 patients (17 women-35 veneers, 18 men-45 veneers) using a standardized clinical procedure during the period between January 2014 and October 2014. The patients were treated with at least one, and at most four veneers. The veneers’ distribution according to the tooth position in the maxilla is summarized in Table 1. Diastema (n = 64), wedge tooth anomalies (n = 9), and re-shaping (n = 7) were the main indications for veneer treatments in the present study. Five recalls were performed, including baseline measurements. All 35 patients that were initially treated with 80 veneer restorations were enrolled for a follow-up examination at baseline, 24, 48, 72, and 84 months.

**Table 1:** Distribution of veneers according to the tooth position in maxilla

Right canine	Right lateral incisor	Right central Incisor	Left central incisor	Left lateral incisor	Left Canine
5	13	22	13	22	5

**Fig. 1:**
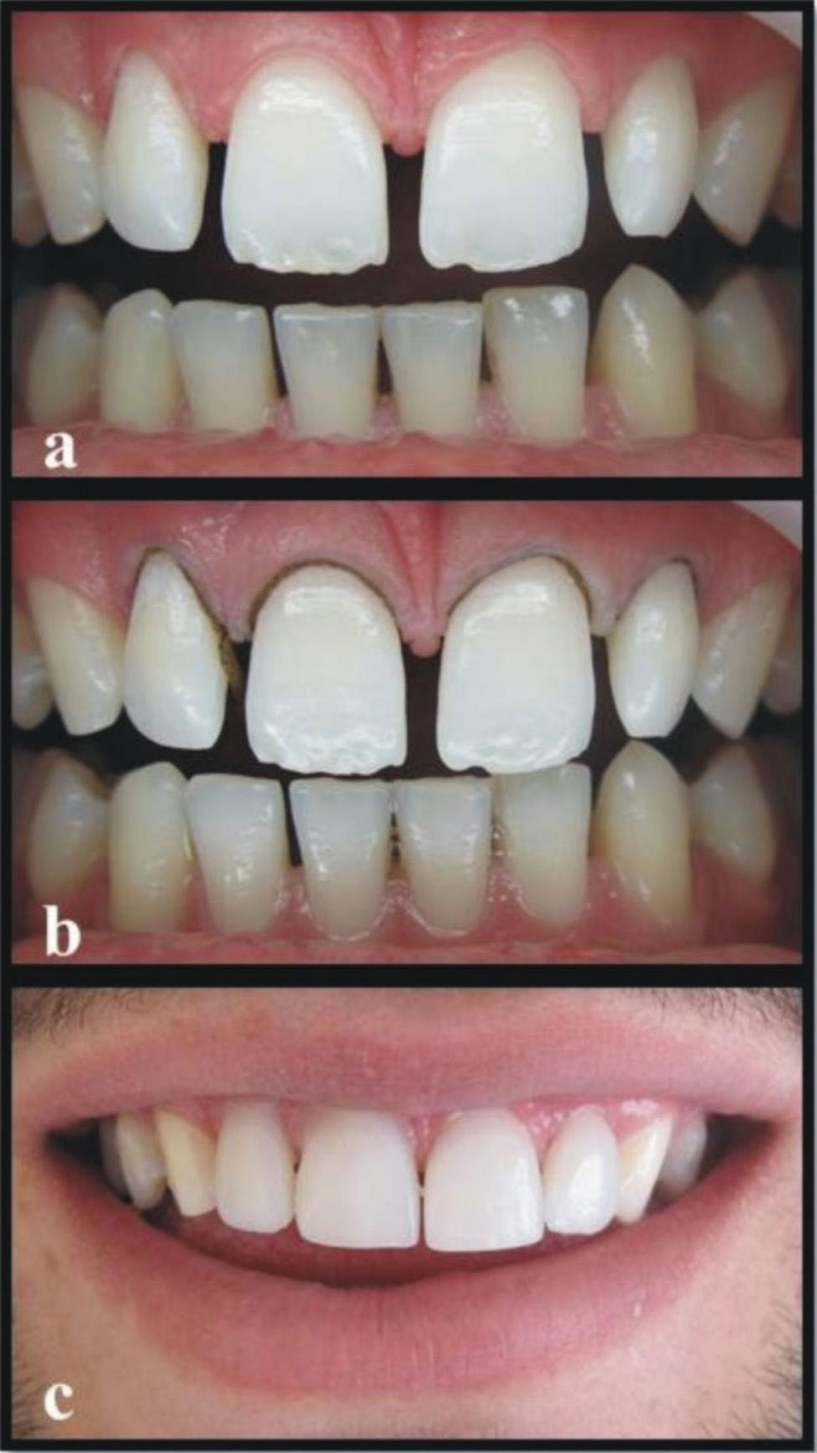
**a.** Preoperative intraoral photograph of patient with diastema between upper incisors. **b.** Intraoral photograph of teeth with retraction cords before cementation. **c.** Postoperative intraoral frontal views of restored incisors after cementation.

**Fig. 2:**
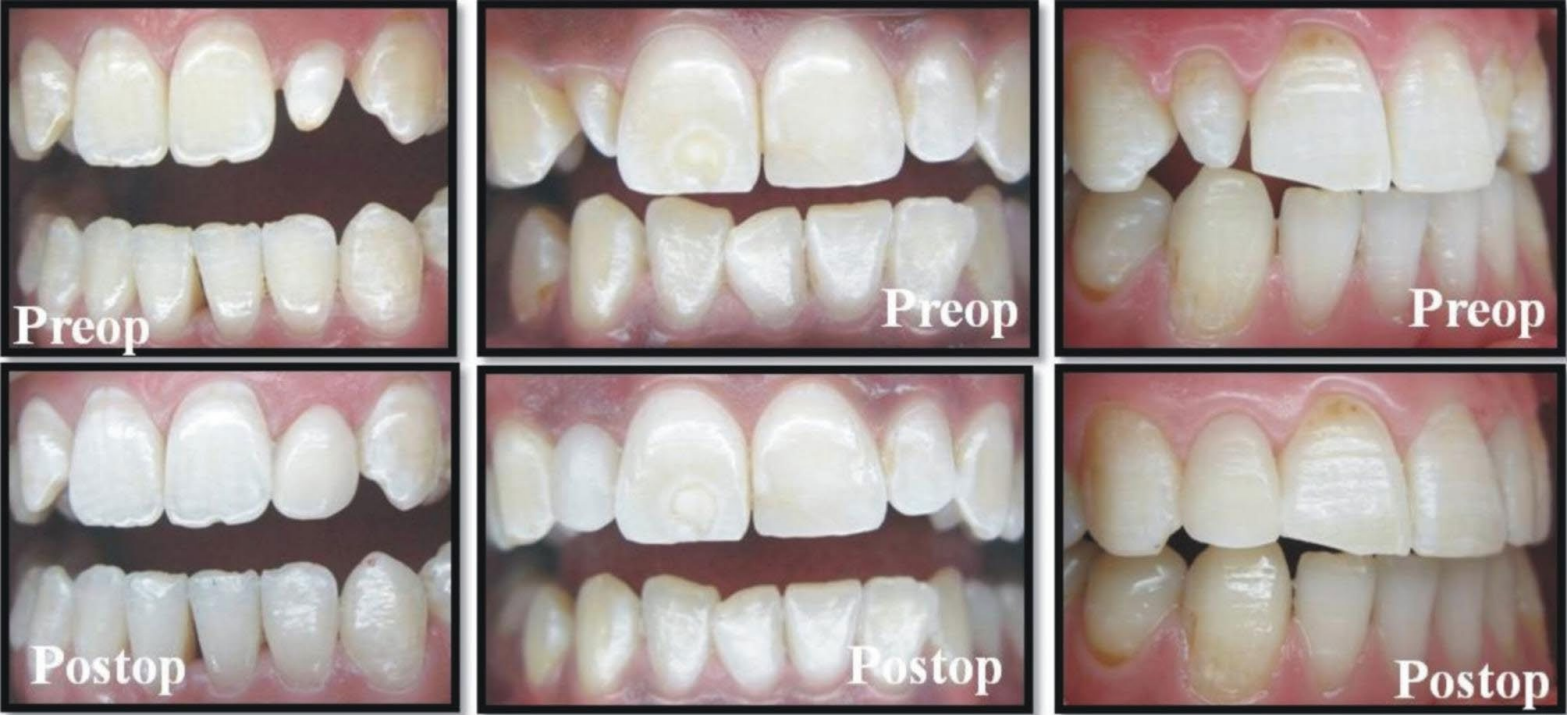
Preoperative and postoperative intraoral photographs of three patients with wedge tooth anomalies

The teeth were cleaned once using a rubber cup with a slurry of pumice water in order to remove any extrinsic stains and dental calculus. Both preoperative and postoperative photographs were taken of each patient (Figs. 1 and 2). The operator controlled the occlusion principles, such as anterior guidance or motion pathways. The teeth were not subjected to any preparation. Retraction cords (Ultrapack, Ultradent, Köln, Germany) were used prior to shade selection, and impression (Fig. 1b). The color selection was completed at the beginning while the teeth were hydrated. Shades were matched with the Vita-Toothguide 3D-Master (Vita Zahnfabrik, Bad Säckingen, Germany) and a Demetron shade light (Kerr Corporation, Middleton, USA). The impression was carried out in two steps, with heavy and light body polyvinyl siloxane impression materials (Elite HD + Putty Soft/Light Body Normal Set, Zhermack, Rovigo, Italy) using a double-mix impression technique. The impression was also used to obtain a master model. The diagnostic wax-up model was prepared, a silicone matrix of this wax-up was produced, and the final shape and position of the teeth was controlled. A type IV dental die stone (GC Fujirock, GC, Leuven, Belgium) was used and then 12 micron die spacer was applied on the casts. The same experienced dental technician fabricated all of the laminate veneers with an indirect micro hybrid composite material (Gradia, GC, IL, USA) using the polychromatic layering technique and with a polymerization oven of the same brand (GC), following the manufacturer’s instructions. At the second visit, the marginal and gingival adaptation, interproximal contact points, shape, and color of the veneers were checked. The teeth were cleaned using a rubber cup with a slurry of pumice water. The operative field was isolated with a rubber dam before the restorative procedures. Dental floss tie was also be used to keep rubberdam clamp in place. Light-cured resin cement (Choice 2, Bisco, Schaumburg, USA) was used to lute the restorations according to the manufacturer’s recommendations. The color shade of the adhesive cement was selected. Each veneer was luted individually. The enamel was etched with 32% phosphoric acid (Uni Etch, Bisco, Schaumburg, USA) for 15 s. After etching, the surface was rinsed and lightly air dried. The A&B parts of the adhesive resin (All-Bond 3, Bisco, Schaumburg, USA) were mixed in a 1:1 ratio and applied 1–2 consecutive coats to enamel. The surface was air dried gently for 10 s to evaporate the solvent and then light cured for 10 s. The inner surfaces of the sandblasted veneers were prepared for cementation. The A&B parts of the adhesive resin (All-Bond 3, Bisco, Schaumburg, USA) were mixed and applied 1–2 consecutive coats to internal surface of the veneer. The surface was air dried gently but not light cured. A generous amount of the selected shade of the cement was applied to the inner surface of the veneers. The veneers were placed on the corresponding teeth using finger pressure. Three to five seconds of light-polymerizing at the incisal edge was performed in order to ensure the veneer’s stabilization and remove excess luting cement. Excess luting cement that extruded from the veneers’ margins was removed with a thin explorer and interproximal floss on the interproximal side. The light polymerization was performed with a halogen curing unit (Demetron LC, Kerr Corporation, Orange, CA, USA) with a light intensity of 550–600 mW/cm^2^ and standard curing mode for 40 s from each tooth’s buccal, incisal, mesial, and distal aspects in accordance with the manufacturer’s instructions. A calibrated radiometer was used to verify the intensity of the light-curing unit after every five patients. The interproximal surfaces were finished with polishing strips (Super-Snap, Shofu, Kyoto, Japan). All patients were recommended to bite hard food carefully after cementation.

In case of multiple restorations, veneers were tested on teeth surfaces alternately and then all together to evaluate the congruence of the proximal contacts. In cementation of side to side veneers, the adjacent veneer was placed after 3–5 s of light polymerization at the incisal edge to stabilize the first placed veneer, and the same procedure was followed for this veneer. The accuracy of interproximal contact points; marginal adaptation; shapes, color and the overall esthetical integration were assessed. After this evaluation, the polymerization process was completed.

### Evaluation procedure

At the baseline recall and at every next annual check-up, clinical performance was assessed in terms of retention, color match, surface roughness, marginal integrity, marginal discoloration, and anatomic form using the modified Modified United States Public Health Service (USPHS) criteria (Table 2) by two independent calibrated clinicians as in previous studies [11, 16]. A dental mirror and probe were used to visually examine the restorations. In cases of disagreement between the clinicians, a consensus was reached through discussion and reevaluation. Each veneer was independently assessed for color match with the adjacent teeth. A sharp probe was used to assess marginal integrity.

**Table 2:** Modified United States Public Health Service (USPHS) criteria used for the clinical evaluations of the restorations

Category	Score	Criteria
Marginal adaptation	0	Smooth margin
	1	All margins closed or possess minor voids or defects (enamel exposed)
	2	Obvious crevice at margin, dentin or base exposed
	3	Debonded from one end
	4	Debonded from both ends
Color match	0	Very good color match
	1	Good color match
	2	Slight mismatch in color or shade
	3	Obvious mismatch, outside the normal range
	4	Gross mismatch
Marginal discoloration	0	No discoloration evident
	1	Slight staining, can be polished away
	2	Obvious staining, cannot be polished away
	3	Gross staining
Surface roughness	0	Smooth surface
	1	Slightly rough or pitted
	2	Rough, cannot be refinished
	3	Surface deeply pitted, irregular grooves
Fracture of restoration	0	No fracture
	1	Minor crack lines over restoration
	2	Minor chippings of restoration (1/4 of restoration)
	3	Moderate chippings of restoration (1/2 of restoration)
	4	Severe chippings (3/4 restoration)
	5	Debonding of restoration
Fracture of tooth	0	No fracture of tooth
	1	Minor crack lines in tooth
	2	Minor chippings of tooth (1/4 of crown)
	3	Moderate chippings of tooth (1/2 of crown)
	4	Crown fracture near cementum enamel line
	5	Crown-root fracture (extraction)
Wear of restoration	0	No wear
	1	Wear
Wear of antagonist	0	No wear
	1	Wear of antagonist
Caries	0	No evidence of caries continuous along the margin of the restoration
	1	Caries evident continuous with the margin of the restoration
Postoperative sensitivity	0	No symptoms
	1	Slight sensitivity
	2	Moderate sensitivity
	3	Severe pain

### Statistical analysis

Survival analyses were performed using a statistical software program (SPSS 23.0; SPSS Inc, Chicago, IL, USA) using the Kaplan-Meier test to obtain the overall survival rate in relation to observation time. Wilcoxon Signed Ranks test was used for significance between the baseline and 84 months scores and between the 24 months and the 84 months scores for USPHS criteria. The statistical analyses were performed at a level of p < 0.05.

## Results

**Fig. 3:**
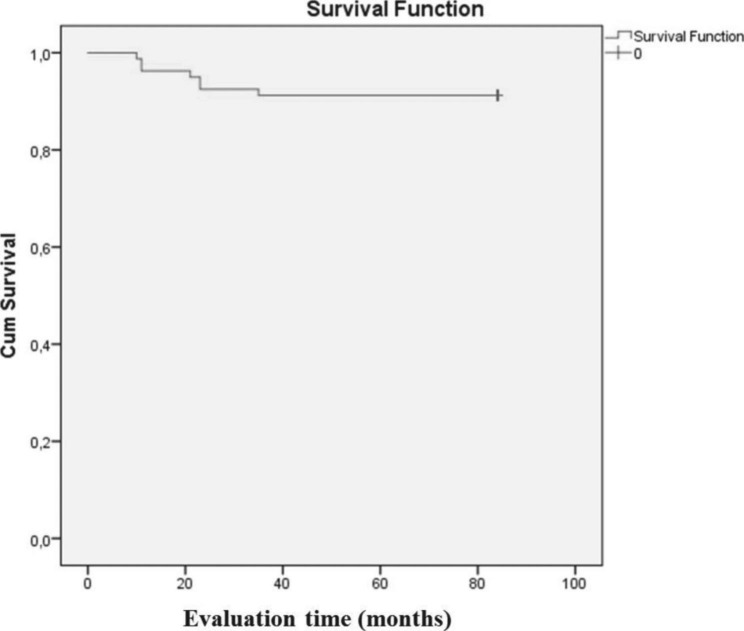
Overall time-dependent survival probability. Cum survival, cumulative survival

The number of restorations examined and the summary of USPHS evaluations in three of the recalls are presented in Table 3. The overall survival rate was 91.3% (Kaplan-Meier) with the mean of retention in the mouth of 78.325 months (Fig. 3) (Table 4). A total of seven failures were observed in the form of debonding (marginal adaptation, score 4) in two patients (n = 4) and fracture (fracture of restoration, score 3) in three patients (n = 3) after 84 months. The first debonding (upper right central incisor, upper left central incisor) failures were observed 11 months after cementation. It was considered a planning error and was not re-cemented.

**Table 3:** The summary of USPHS evaluations in three of the recalls

Category	Score	Baseline n = 80	24 months n = 74	84 months n = 73
Marginal adaptation	0	80	70	66
	1	-	4	7
	2	-	-	-
	3	-	-	-
	4	-	4	4
Color match	0	80	62	24
	1	-	12	34
	2	-	-	15
	3	-	-	-
	4	-	-	-
Marginal discoloration	0	80	70	58
	1	-	4	15
	2	-	-	-
	3	-	-	-
Surface roughness	0	80	66	32
	1	-	8	41
	2	-	-	-
	3	-	-	-
Fracture of restoration	0	80	74	63
	1	-	-	-
	2	-	-	10
	3	-	2	3
	4	-	-	-
	5	-	-	-
Fracture of tooth	0	80	74	73
	1	-	-	-
	2	-	-	-
	3	-	-	-
	4	-	-	-
	5	-	-	-
Wear of restoration	0	80	74	73
	1	-		
Wear of antagonist	0	80	74	73
	1	-	-	-
Caries	0	80	74	73
	1	-	-	-
Postoperative sensitivity	0	80	74	73
	1	-	-	-
	2	-	-	-
	3	-	-	-

**Table 4:** Mean values for survival time

Mean			
Estimate	Std. Error	95% Confidence Interval	
		Lower Bound	Upper Bound
78.325	2.068	74.272	82.378

The other debonding failures (upper right central incisor, upper left central incisor) between the veneer-tooth interfaces were observed 23 months after cementation. After the adhesive surface was cleaned, the debonded veneers were re-adhered, but the patient declared that he did not want to come to other controls for these veneers. The veneers were not evaluated further, and were scored as a failure. Since four teeth with debonding were observed in two different patients in the study, there were 33 numbers of patients who came to the controls at the end of 24 months. Apart from these debonding cases, while there were four veneers that scored as one in the marginal adaptation category in 24 months, this number increased to only seven in 84 months. No procedure was applied to these slight marginal defects.

**Fig. 4:**
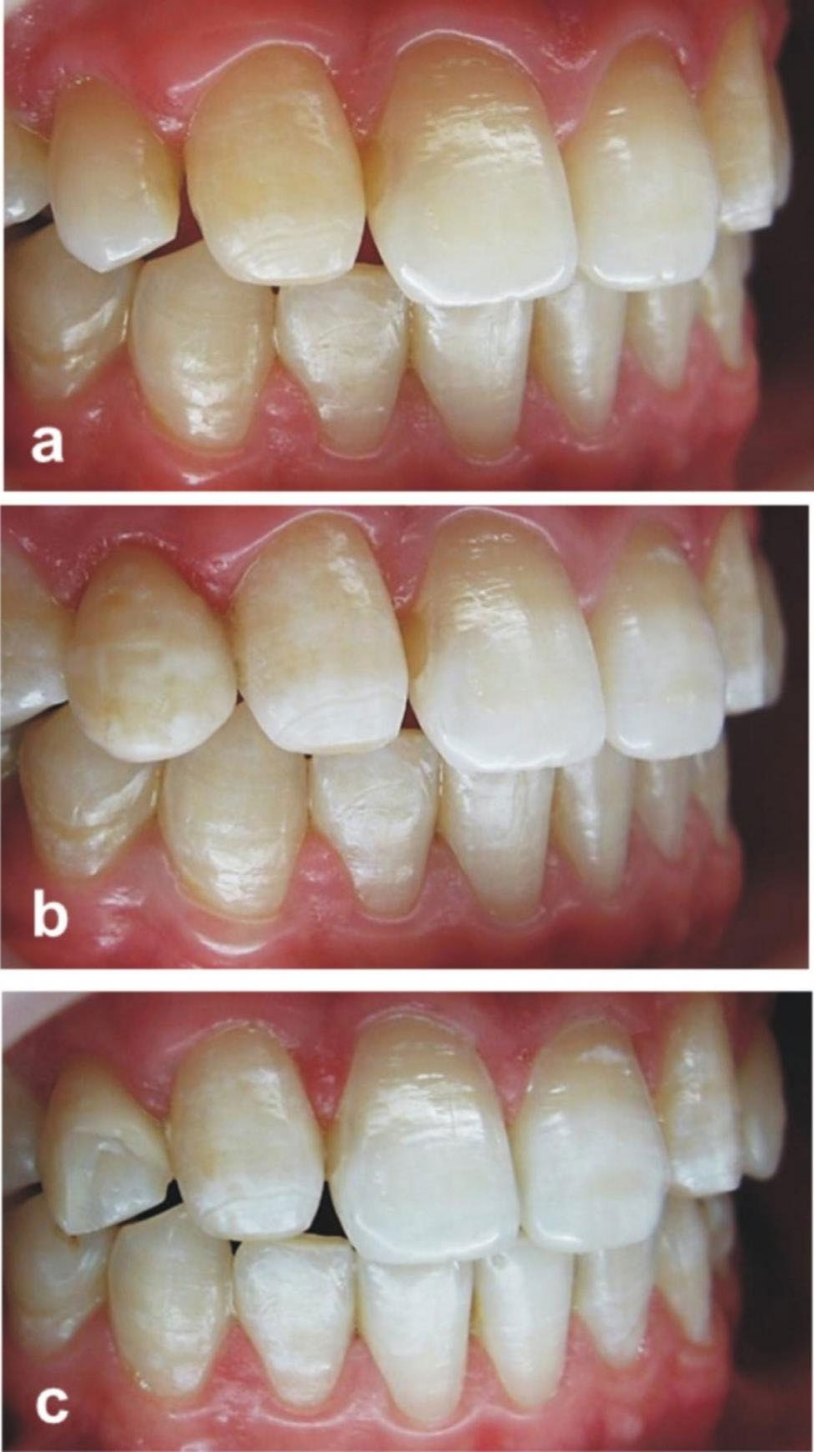
**a.** Preoperative photograph showing a problem of shape of the upper right canine. **b.** Postoperative photograph after the cementation of the composite laminate veneer on upper right canine.**c.** The photograph of the fracture of the composite laminate veneer on upper right canine due to trauma

In the fracture of restoration category, there were three veneers that scored as three. The first fracture occurred on upper right canine, 10 months after luting in a re-shaping case (Fig. 4). The second laminate fracture occurred on upper right lateral incisor, 21 months after luting. The third fracture occurred on tooth number upper right lateral incisor, 35 months after placement. The second and third fractures were observed in diastema cases. All of the patients with these fractures reported that they bit very hard food, despite being warned against doing so. The detached fragment was repaired with a different adhesive system and composite resin. Since these teeth were repaired with a different material, they were evaluated as score three after 84 months. Ten veneers showed minimal composite fractures (chippings) in the incisal and proximal areas. All of these fractures were observed in cases of diastema. While the number of veneers included in the study was 74 at the end of 24 months, it decreased to 73 in 33 patients at the end of 84 months.

**Fig. 5:**
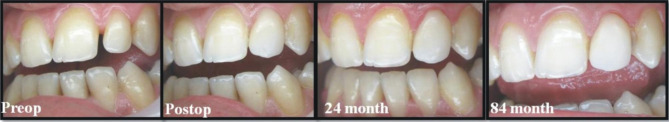
Intraoral photographs of patient with diastema in left lateral incisor at preop, postop, 24-months and 84-months follow-ups

Color match was scored as one (n = 12) during the 24 months follow-up period, without recording any score increase. It was observed that this change occurred due to the loss of gloss in the composite resin in general (Fig. 5). However, there was an increase in the number of laminate veneers scored as one (n = 34) and two (n = 15) during the 84 months evaluation period.

**Fig. 6:**
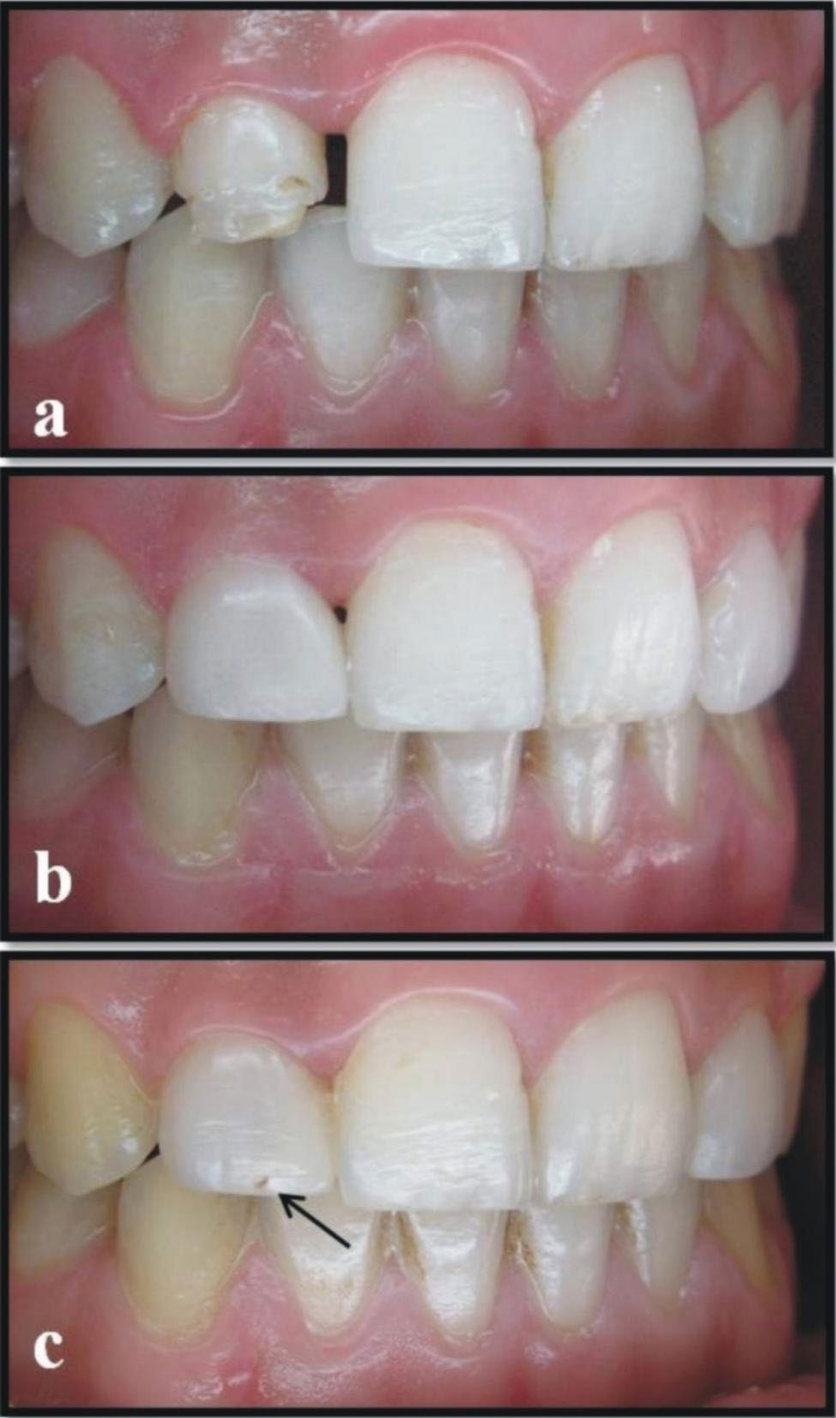
**a.** Preoperative photograph showing a problem of shape of the upper right lateral. **b.** Postoperative photograph after the cementation of the composite laminate veneer on upper right lateral. **c.** The photograph of slightly rough and pitted surface of laminate veneer (Surface roughness, score 1) on upper right lateral at 24 months follow-up

While slightly rough surfaces (Surface roughness-score 1) were observed in eight laminate veneers at 24 months (Fig. 6) and in 41 laminate veneers at 84 months. Slight marginal discolorations were noted in four laminates during the 24 months recall and by the last recall the number of slight marginal discolorations increased to 15 across 73 laminates. All of these discolorations were eliminated by polishing discs (Super-Snap, Shofu, Kyoto, Japan). Wear of restoration, wear of antagonist, secondary caries, and postoperative sensitivity were not observed in any of the cases.

The Wilcoxon Signed Ranks test results (p-values) after comparing the baseline-84 months scores and the 24 months-84 months scores for each USPHS criterion are presented in Table 5. Since the baseline, 24 months and 84 months results were constant for wear of restoration, wear of antagonist, and secondary caries criteria, no statistics were calculated.

For the fracture of teeth and postoperative sensitivity criteria, no statistically significant differences were found both between baseline-84 months and 24 months-84 months scores (p = 1.000). The marginal adaptation scores between 24 months and 84 months were not statistically significant (p = 0.165). The overall scores after 84 months were significantly higher than the baseline scores for the marginal adaptation (p = 0.008), color match (p = 0.000), marginal discoloration (p = 0.000), surface roughness (p = 0.000), and fracture of restoration (p = 0.001) criteria. The scores after 84 months were significantly higher than the 24 months scores for the criteria of color match (p = 0.000), marginal discoloration (p = 0.001), surface roughness (p = 0.000), fracture of restoration (p = 0.001), respectively.

**Table 5:** Wilcoxon Signed Ranks test results (p-values) comparing the baseline, 24 months and 84 months scores for USPHS criteria

	p-values	
**Category**	Baseline-84 months	24 months-84 months
Marginal adaptation	0.008*	0.165
Color match	0.000*	0.000*
Marginal discoloration	0.000*	0.001*
Surface roughness	0.000*	0.000*
Fracture of restoration	0.001*	0.001*
Fracture of tooth	1.000	1.000
Postoperative sensitivity	1.000	1.000

*p < 0.05.

## Discussion

Current guidelines emphasize the importance of developing minimally invasive treatments that preserve as much tooth tissue as possible, and this preservation is now possible thanks to the development of adhesive dentistry [13]. Considering this tendency towards more conservative preparations and bonding procedures, laminate veneers have become one of the most preferred treatment modalities [8]. In laminate veneers, the no-prep technique is an excellent rehabilitation option in cases where the dental tissues are healthy and can be changed by adding material only. The idea behind the no-prep technique is to restore the tooth’s function and appearance avoiding unnecessary wear and tear on the tooth structure and applying the most conservative method possible for the clinician [7]. Furthermore, this approach ensures the clinician will control the invasion of the gingival groove, position the prosthetic termination line at different levels within the groove, allow the optimal restoration-tooth relationship, and correct the cementoenamel junction on non-prepared teeth [17].

Objective and reliable criteria should be used to determine the performance of restorative materials in clinical trials. The USPHS criteria are widely preferred in clinical follow-up studies because they are easy to apply and provide an opportunity for the results to be compared with previous studies [8, 18, 19]. Therefore, as in many previous clinical studies, modified USPHS criteria were used in this clinical follow-up study to evaluate the clinical performance of indirect composite veneers without a preparation technique [11, 16, 20].

Until today, ceramic has been used more widely among different materials to fabricate laminate veneers and literature is more extensive on this topic for ceramic than composite resin [16, 21–23]. There are many studies reporting that ceramic veneers perform better than indirect composite laminate veneers [7, 24]. However, it was also reported that composite laminate veneers have gained great importance as they could meet the increasing esthetic demand and provide the patient with minimal prepared or unprepared treatment options [5]. The fact that no preparation is made in this technique requires some criteria to be considered in selecting materials. When planning this in vivo study, many factors were taken into account in deciding whether the type of material to be used should be composite resin. One of the most important issues to consider is the need for tooth preparation when using ceramic in laminate veneer restorations. Some authors believe that the technique for laminate veneers requires a shallow reduction of the enamel on the labial surface because of the strength, seat, and color of ceramic/porcelain [25]. In a previous study, although not evidence-based, it was reported that preparations for porcelain laminate veneers require the operator to reduce the labial surface by 0.5 mm uniformly within the enamel [26]. In a study concerning a systematic review and meta-analysis of survival and complication rates of ceramic and porcelain reported that various preparation depths and methods have been defined for porcelain laminate veneers [8]. However, some dental ceramic manufacturers offer deep preparations in order to increase the ceramics’ strength [27].

Another criteria to consider is that no-prep veneers require superior skills in the laboratory, as ultrathin veneers can be particularly difficult to manufacture and process [6]. According to D’Arcangelo et al., the very brittle nature of thin feldspathic veneers, which can easily crack during fabrication and placement, was the main limitation for these restorations [6]. However, they noted that thin porcelain shells could be particularly challenging to apply to unprepared teeth, necessitating the use of very fine resin composite cement in order to prevent bending forces during seating [6]. Composites have no potential for catastrophic brittle fracture and do not cause abrasive wear on opposing teeth compared to ceramic restorations [10]. In addition, compared to other restorative materials, single-session, intraoral repair is possible in composite restorations without the risk of altering esthetics or mechanical performance [10]. It should not be forgotten that composite veneer is an economical option to ceramic veneer with comparable esthetic, physical, and optical properties [3].

The fact that the absence of tooth preparation and all margins of laminate veneers are placed on enamel using no-prep technique provided many favorable outcomes in this study. One of the most important outcomes was the high survival rate of the laminate veneers: 91.3%. Based on the present data, the null hypothesis of this study was rejected. While there is a consensus about the predictability of traditional veneers bonded to prepared teeth, there are limited studies on the clinical performance of laminate veneers placed without tooth preparation. [8, 28, 29]. In previous studies, composite laminate veneers with tooth preparation showed survival rates of 87% and 75% after 3 years and 10 years, respectively [11, 24]. The high rate of composite laminate veneers in this study may be due to the conservative approach of preserving dental structure, especially enamel. Conservation of enamel gains importance in terms of the adhesion mechanism. It is a well-known fact that an optimal bond is achieved if the preparation is conducted completely in enamel [30]. This optimal bond provides the tooth and restoration act as a whole, optimizing long-term durability and strength [17]. A recent study evaluating the performance of no-prep porcelain veneers also reported a high overall survival rate of 97.4% after 36 to 60 months of clinical service [28]. In a 2-year clinical study comparing porcelain laminate veneers with minimal tooth preparation and those with no preparation, where enamel preservation was achieved, the survival rate in both cases was 100% [31]. Morimoto et al. reported that bonding to enamel provides a decrease in marginal discoloration, debonding, chipping, and fractures [8]. They also reported that the larger the amount of tooth preserved, the smaller the deflexion of the tooth, and this might explain the low failure rates [8].

However, considering these advances in materials and techniques, it is thought that the other clinical factors may be responsible for early debonding failures (only four debonding out of 80 laminate veneers) in this study. Early failures may result from incorrect treatment planning, technical faults, or patient-dependent factors. These results demonstrated the fact that the success of a clinical procedure depends on the indication, planning, clinical and laboratory steps, and patient habits [8]. Two debonding failures in the present study occurred on both central incisors in the same patient within the first year following luting. This failure may be attributed to the lack of versatile evaluation in treatment planning. In this respect, the requirement for the dentist to complete dental and esthetic analysis in full including an assessment of the patient’s wishes and expectations; the analysis of radiographs, photographs, and mounted models; and periodontal examination before advising any patient about the treatment options has once again gained importance [14]. Therefore, dental analysis, such as occlusion, midline and incisal edge position, facial profile, lip fullness, the shapes of the teeth, and the desired color change should be evaluated together for an appropriate treatment plan through the clinician’s experience and knowledge [6, 14]. De Angelis et al. also reported that the previous poor results observed with no prep veneers might be attributed to the lack of guidelines for technical procedures and patient selection, rather than the technique itself [28]. The other two debonding failures occurred in the same patient on both central incisors 23 months after placement. Although the patient stated that there was no bruxism in anamnesis and there was no sign of bruxism in the clinical examination, she stated in the control sessions that she noticed she clenched her teeth excessively as the result of daytime stress and nervousness. Therefore, these failures may have developed due to uncontrolled occlusal stresses. These uncontrolled non-functional jaw movements are recognized as bruxism. Bruxism is a diurnal or nocturnal parafunctional activity involving forceful clenching or grinding of the teeth, or a combination of both. In these cases, the adhesion between resin and luting resin is affected, thereby reducing the bond strength between the veneer-tooth complexes [32].

The absence of postoperative sensitivity, secondary caries, wear of antagonist and tooth fracture, and a low rate of marginal adaptation scores were the other favorable outcomes of this technique in this study. Without tooth preparation, the shape of the teeth remains unchanged, and periodontal maintenance and marginal adaptations are more sustainable, thus eliminating the need for temporary restoration and sensitivity problems [17]. Many authors have suggested that restoration margins are affected by preparation, cementation, and finishing procedures [10, 30]. The lack of preparation and need for finishing may be an indicator of the success of marginal adaptation in the present study with no significant difference between the results of 24 months and 84 months (p = 0.165). In this case, the importance of the cementation procedure and luting cement in no-prep laminate veneers becomes obvious. Therefore, the choice of luting cement requires more attention in no-prep techniques. In this study, a light-cured cement was used, as in many previous studies [7, 10, 11, 30]. The working time required to position the indirect restorations and remove excess cement was appropriately extended at the clinician’s discretion using light-curing composite rather than dual-cure cements, such as the luting agent. However, diamond burs, or finishing discs, were not used to finish the restorations in order to avoid affecting the marginal adaptation. Residual cement was only removed with an explorer, scalpel, dental floss, and, where necessary, interproximal polishing discs, as in some other studies [10]. De Angelis et al. also used only hand instruments in the removal of adhesive and excess cement in their study and reported that this was one of the factors affecting the marginal quality [28].

While the use of light-cured resin cements is a satisfactory choice for luting veneers and increasing the esthetic quality of restorations as well as retention and fracture resistance, appropriate photo-activation of the materials used in the luting procedure is also an essential step to ensure the esthetic, marginal and mechanical longevity of laminate restorations [33]. The energy density provided by the light-curing unit may have a significant effect on the polymerization kinetics and the degree of polymerization [34]. It was previously stated that the degree of polymerization is related to the total amount of energy absorbed by the resin, and the total light energy is related to the intensity of the light and the exposure time. The use of light-emitting diodes (LEDs) has been reported to produce more intense light, which can mean shorter curing time and higher bond strength [35]. In this study, a halogen curing unit was used for luting the resin cement. Although there are studies using a halogen curing unit in the luting of resin cements [30], LEDs are more commonly used for luting veneers considering up-to-date clinical luting approaches [17, 36]. Although low marginal adaptation scores were observed, the low bond strength of resin cements due to the use of halogen may be considered as a factor affecting marginal adaptation and as a reason for this result. In this case, the use of halogen light device in our study can be regarded as a limitation of the study.

Although composite resins have excellent esthetic properties, their susceptibility to discoloration after prolonged exposure to the oral environment is a major drawback. Unacceptable color change, particularly marginal discoloration, appears to be the primary reason for replacing composite restorations [37]. The results of this study showed that the most common failures were color match and surface roughness, confirming previous findings [11, 24]. This situation also appeared as a statistically significant difference both between baseline-84 months and 24 months-84 months results in color match (p = 0.000) and surface roughness (p = 0.000) criteria. A published clinical trial (with a 3-years follow-up) has reported no significant difference in survival rates for composite (87%) and ceramic (100%) veneers; however, some surface quality changes were observed more frequently in composite resin materials, such as small voids, defects, and slight marginal staining [11]. Similarly, the most frequently observed changes in the present study were slight surface degradation manifested by minor voids and decreased gloss retention of the indirect resin composite material. Short-term color stability due to pigmentation and low abrasion resistance due to loss of gloss and texture are two of the expected results in composite resins [7]. In this case, reasons for marginal discoloration of indirect composite restorations may be attributed to the loss of luting cement in this region due to wear or the accumulation of coloring materials due to surface degradation. Coloring materials and smoking may be a problem in terms of staining if composite restorations are used for patients who smoke heavily [21].

The placement of all margins of the laminate veneers on the enamel and their complete adherence to the enamel positively affected the early marginal discoloration results and very low scores were obtained at 24 months. In a retrospective clinical trial on no-prep porcelain veneers, De Angelis et al. also reported low percentage of marginal discoloration (2.6%) after 36 to 60 months of clinical service. They stated that these discolorations were not noticed or perceived as a defect by patients and were classified as relative failures [28]. But in this study, re-polishing was applied to 4 veneers with marginal discoloration score of 1. No discoloration was observed in re-polished veneers at the 84-month follow-up. However, marginal discoloration of the restorations increased with time in this study, consistent with a previous study [29]. Arif et al. reported that marginal discoloration increased with the increasing age of the restorations [29]. They also stated that thermal contractions of composite resins could lead to deformation and cracks by creating stresses at the margins [29]. In addition to the factors mentioned above, time-dependent surface quality changes of the composite resin were thought to be effective in statistically significant increase after 24 months (p = 0.001).

The remarkable thing in this study was that all of the minor fractures or chippings were seen in diastema cases involving the anterior four teeth at the incisal aspect. It has been previously stated that very low thickness veneers can be used in diastema cases without any preparation technique [6]. However, these results showed the necessity that the diastema width be within a certain range, which is also a limitation of this study. This approach can be challenging in terms of material resistance in very wide diastemas. In a study evaluating the 7-year clinical outcomes of feldspathic ceramic laminate veneers using minimally invasive techniques such as vertical preparation or no-prep, the authors concluded that when there are large unsupported feldspathic ceramic areas (such as in diastema closure and fractured teeth), the ceramic material needs to be extended more than 2 mm from the surface of the tooth resulting higher tensile and shear stresses due to the weakening of these materials. They also reported that the ceramic is susceptible to cracks in gaps larger than 1.5 mm [38]. This situation increases the importance given to luting cement and bond strength. It has been previously stated that, for an esthetic outcome, the resin cement must be of minimum thickness at the interface [21]. It has been previously stated that polymerization shrinkage of the luting cement can create stress concentrations at the adhesive interface or that failures can occur simply due to heavy occlusion [9]. It should also be noted that the thickness of the luting cement also has a significant effect on the stress distribution. Thin laminate veneers with poor internal fit have been reported to cause higher stresses, both at the interface of the restoration and at the surface, thereby causing post-bonding cracks or chipping in thin laminate veneers [6]. In another study, such failures were attributed to the presence of unsupported areas under the composite veneer or to the cohesive strength of the composite itself [39].

The other limitation of this study was the lack of a comparison group in the present case series study. Another factor to consider is that the study protocol included less challenging conditions in the study and more challenging cases could also be included.

## Conclusion

Within its methodological limitations, the following conclusions might be drawn in the present study:

- No-prep veneers showed biologically healthy and compatible marginal edges. After a longtime evaluation period, the overall outcome and clinical acceptance were satisfactory.
- This technique preserves the whole natural tooth substance, but appropriate patient selection, adhesive principles, laboratory experience, and cementation techniques must be properly selected and managed in order to ensure success.
- Furthermore, clinical trials should be conducted on the long-term efficacy of different materials with the no-prep technique for anterior veneer restorations.

## Data Availability

The datasets generated and/or analysed during the current study are not publicly available due to patients’ privacy but are available from the corresponding author on reasonable request.
